# Increased variability in reaction time is associated with amyloid beta pathology at age 70

**DOI:** 10.1002/dad2.12076

**Published:** 2020-08-10

**Authors:** Kirsty Lu, Jennifer M. Nicholas, Sarah‐Naomi James, Christopher A. Lane, Thomas D. Parker, Ashvini Keshavan, Sarah E. Keuss, Sarah M. Buchanan, Heidi Murray‐Smith, David M. Cash, Carole H. Sudre, Ian B. Malone, William Coath, Andrew Wong, Susie M.D. Henley, Nick C. Fox, Marcus Richards, Jonathan M. Schott, Sebastian J. Crutch

**Affiliations:** ^1^ Dementia Research Centre UCL Queen Square Institute of Neurology, University College London London UK; ^2^ Department of Medical Statistics London School of Hygiene and Tropical Medicine London UK; ^3^ MRC Unit for Lifelong Health and Ageing at UCL University College London London UK; ^4^ School of Biomedical Engineering and Imaging Sciences King's College London London UK; ^5^ Department of Medical Physics University College London London UK; ^6^ UK Dementia Research Institute at University College London London UK

**Keywords:** amyloid‐positron emission tomography imaging, birth cohort, cognition, cognitive decline, dementia, healthy aging, preclinical Alzheimer's disease, reaction time, variability

## Abstract

**Introduction:**

We investigated whether life‐course factors and neuroimaging biomarkers of Alzheimer's disease pathology predict reaction time (RT) performance in older adults.

**Methods:**

Insight 46 study participants, all born in the same week in 1946 (n = 501; ages at assessment = 69 to 71 years), completed a 2‐choice RT task and amyloid beta (Aβ) positron emission tomography and MR imaging. We tested for associations between task outcomes (RT; error rate; intra‐individual variability in RT) and life‐course predictors including childhood cognitive ability and education. In a subsample of 406 cognitively normal participants, we investigated associations between task outcomes and biomarkers including Aβ‐positivity.

**Results:**

Cognitively normal Aβ‐positive participants had 10% more variable RTs than Aβ‐negative participants, despite having similar mean RTs. Childhood cognitive ability and education independently predicted task performance.

**Discussion:**

This study provides novel evidence that Aβ pathology is associated with poorer consistency of RT in cognitively normal older adults, at an age when dementia prevalence is still very low.

## 1 BACKGROUND

Many cognitively normal older adults show biomarker evidence of accumulating brain pathologies in an age‐related manner, with a significant proportion meeting criteria for preclinical Alzheimer's disease (AD).^1^, ^2^ As the preclinical stage of AD extends up to around 20 years before the onset of symptoms,^3^, ^4^ and many individuals meeting the biomarker criteria may never develop cognitive impairment within their lifetimes,^5^ it is important to understand the relationship between preclinical pathology and cognitive decline. While there is evidence that subtle cognitive decline is detectable in older adults with preclinical AD,^6^, ^7^, ^8^ questions remain about the nature and timing of the earliest cognitive changes and how they may be disentangled from age‐related cognitive decline. Answering these questions would improve our ability to identify individuals at highest risk of cognitive impairment, as well as informing the selection of sensitive cognitive measures for clinical trials.

Throughout adulthood, reaction time (RT) becomes notably slower and less consistent (ie, intra‐individual variability [IIV] increases).^9^, ^10^, ^11^, ^12^, ^13^, ^14^, ^15^ Patients with AD are reported to have slower RTs and greater IIV than controls,^15^, ^16^, ^17^, ^18^ with a similar pattern observed in mild cognitive impairment (MCI).^16^, ^19^, ^20^, ^21^, ^22^ Choice reaction time (CRT, requiring selection of the correct response from two or more options) may be particularly informative because—compared to simple RT—it is more sensitive to age and cerebral dysfunction.^10^, ^13^ To our knowledge, no studies have investigated associations between CRT performance (RT, IIV, and error rate) and biomarkers of AD pathology in cognitively normal older adults. There is some evidence that individuals meeting criteria for preclinical AD are subtly impaired on paper‐and‐pencil measures of processing speed (eg, Baker et al. ^6^ and Ho and Nation^8^), but such tests do not allow measurement of IIV.

If subtle cognitive decline in the preclinical phase of AD is to be detected, it is important to account for other factors that may contribute to inter‐individual differences, such as sex and prior cognitive ability. Evidence for sex differences in RT is mixed,^10^, ^11^, ^16^, ^19^, ^23^ whereas associations between RT and general cognitive ability are well established.^9^, ^24^, ^25^, ^26^, ^27^ While CRT experiments typically consider RT separately from error rate, it may also be important to consider speed‐accuracy trade‐offs.

Insight 46, a substudy of the National Survey of Health and Development (NSHD, the British 1946 Birth Cohort), provides a unique opportunity to study preclinical AD. Participants are drawn from the world's longest continuously running birth cohort study, with life‐course data available on their cognition since childhood as well as a wealth of sociodemographic, health, and lifestyle factors.^28^ They were assessed at age ≈70 years, when the prevalence of dementia is low (≈3%)^29^ but the prevalence of amyloid beta (Aβ) pathology is already around 15% to 25%.^2^, ^30^ We recently reported that cognitively normal Aβ‐positive participants scored slightly lower than Aβ‐negative participants on standard paper‐and‐pencil measures of episodic memory, processing speed, and non‐verbal reasoning.^31^ We now report the results of a CRT task.

The aims of this study were to: (1) describe performance on a CRT task and its associations with demographic and life‐course predictors; and (2) investigate associations between task performance and biomarkers of brain pathologies among cognitively normal participants. Our key hypothesis was that Aβ‐positive participants would have slower RTs and greater IIV than Aβ‐negative participants.

**HIGHLIGHTS**

- Participants (n = 501) were all born in the same week in March 1946.
- Assessments at age ∼70 included a choice reaction time test and amyloid‐PET/MRI.
- Amyloid pathology was associated with greater variability in reaction time.
- Reaction time variability may be a sensitive measure of subtle cognitive decline.

**RESEARCH IN CONTEXT**

1. Systematic review: The authors reviewed the literature using traditional sources (eg, PubMed). While there is evidence that subtle cognitive decline is detectable during the preclinical stage of Alzheimer's disease, the nature of changes in reaction time has received little attention.
2. Interpretation: In a sample of British adults all born during the same week in 1946, cognitively normal individuals with amyloid beta (Aβ) pathology were less consistent in their response times on a choice reaction time (CRT) task, although their responses were not slower overall. This builds on previous evidence that Aβ pathology is associated with subtle changes in multiple cognitive domains (ie, not just memory) by age 70.
3. Future Directions: Continued follow‐up of these participants will be important to examine how changes in CRT relate to accumulating pathologies. Further studies would be beneficial to examine the potential of CRT tasks as sensitive measures of subtle cognitive decline for clinical trials.

## 2 METHODS

The NSHD is a population‐based sample originally consisting of 5362 individuals born across mainland Britain during one week in March 1946, who have been assessed 24 times across the lifespan.^28^ Recruitment procedures, assessment protocols, and recruitment flow‐charts for the Insight 46 substudy have been previously published.^31^, ^32^, ^33^, ^34^ In brief, 502 individuals attended University College London between May 2015 and January 2018 and underwent cognitive tests, clinical examination, brain magnetic resonance imaging (MRI), Aβ‐positron emission tomography (PET), and other biomarker measures. The study was approved by the National Research Ethics Service Committee London (REC reference 14/LO/1173). Participants provided written informed consent.

### 2.1 CRT task

#### 2.1.1 Procedure

**FIGURE 1:**
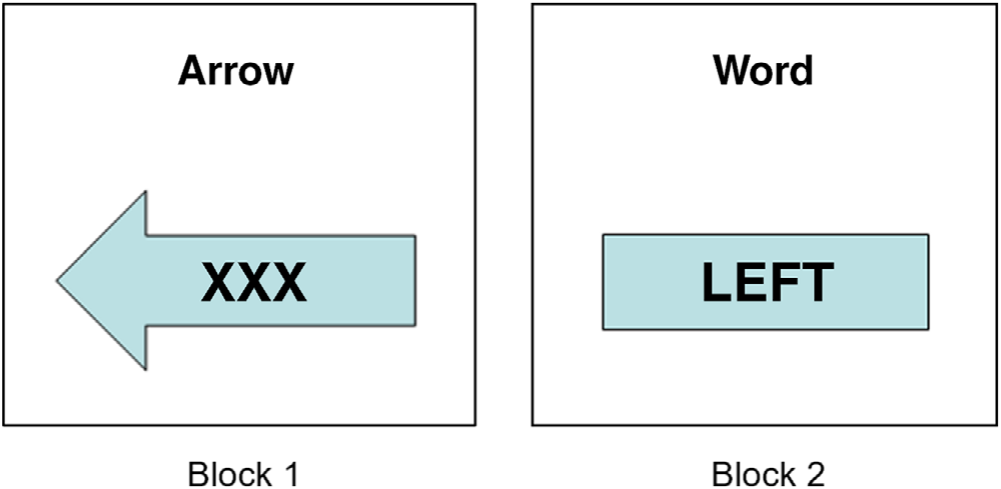
The choice reaction time task. The cue (“Arrow" in Block 1; “Word" in Block 2) appeared on its own for 1000 ms before the stimulus appeared underneath. The XXX inside the arrow is not relevant for the response, but was included so that the appearance and visual complexity of both blocks were as similar as possible

The CRT task was inspired by previous experiments.^35^, ^36^ Stimuli were presented on a DELL Optiplex 9030 computer, using SuperLab software. A Cedrus RB‐740 response box with two buttons side by side was placed in front of the participant. The experiment had two blocks: in block 1 the stimulus was an arrow pointing left or right; in block 2 the stimulus was the word ‘LEFT’ or ‘RIGHT’ (Figure 1). All stimuli were displayed in the center of the screen.

Each block contained 12 trials in a fixed order (Block 1: LRRRLRLRLLRL; Block 2: LLLLRRRLRRRL). To pace the experiment, each trial began with a cue displayed for 1000 ms (“Arrow" in Block 1; “Word" in Block 2), before the stimulus appeared. Six practice trials preceded each block.

Participants were instructed to press the correct button (left or right) as quickly as possible, using the index and middle fingers of their dominant hand. If their response was correct, the next trial was initiated; if incorrect, the stimulus remained on the screen and an error tone signaled that they should respond again. Regardless of whether their second attempt was correct or incorrect, no feedback was given and the next trial was initiated.

Two variables were recorded for each response: RT (ms) and accuracy (correct or incorrect). The total time to complete the task—including explaining the instructions and completing the practice—was extracted from the output.

#### 2.1.2 Data processing

During testing it was observed that participants were not always ready for the first trial of each block, so these trials were dropped from analysis. All analyses were based on the initial response to each stimulus; second attempts were not analyzed as their purpose was to reorient participants to the task. As the highest error rate was 27% (Table 1), no participants were excluded based on responding at random. RTs were checked for outliers. As in a previous study,^35^ a threshold of 300 ms was adopted as the minimum time for a valid response, although in fact no responses were faster than this. A threshold of three standard deviations (SD) above each participant's own mean was used to exclude outlying slow responses,^12^, ^37^ resulting in the exclusion of 0.4% of responses from the RT analyses.

**TABLE 1:** Participant characteristics and descriptive statistics for the choice reaction time task (n = 501)

		Cognitively normal^a^ participants with complete biomarker data (n = 406)^b^	
	All participants	Aβ positive	Aβ negative
N	501	74	332
Sex: % female	49	46	51
Age at assessment (years)^c^: mean, SD, (range)	70.7, 0.68 (69.2 to 71.8)	70.6, 0.66 (69.4 to 71.8)	70.6, 0.70 (69.2 to 71.8)
Highest educational qualification: %			
None	15.6	17.6	15.4
Below O‐levels (vocational)	5.2	6.8	4.2
O‐levels or equivalent	24.8	25.7	26.2
A‐levels or equivalent	35.7	32.4	35.2
Degree or equivalent	18.8	17.6	19.0
Childhood cognitive ability (z‐score)^d^: mean, SD, (range)	0.39, 0.74 (−1.60 to 2.50)	0.44, 0.74 (−1.37, 2.50)	0.41, 0.74 (−1.59 to 2.47)
Socioeconomic position: %			
Unskilled	1.0	1.4	0.6
Partially skilled	4.8	2.7	5.4
Skilled manual	9.4	9.5	9.3
Skilled non‐manual	21.2	16.2	22.0
Intermediate	52.3	55.4	51.8
Professional	11.4	14.9	10.8
MMSE score (max. 30): median, IQR, (range)	30, 30 to 30 (22 to 30)	29, 29 to 30 (26 to 30)	30, 30 to 30 (26 to 30)
Standard uptake volume ratio: median, IQR, (range)	0.55, 0.51 to 0.58 (0.45 to 0.87)^e^	0.67, 0.64 to 0.71 (0.61 to 0.87)	0.53, 0.51 to 0.56 (0.47 to 0.61)
White matter hyperintensity volume (cm^3^): median, IQR, (range)	3.1, 1.6 to 6.8 (0.3 to 33.7)^f^	3.3, 1.8 to 6.8 (0.3 to 33.7)	2.9, 1.5 to 6.4 (0.3 to 32.8)
Whole brain volume (cm^3^): mean, SD, (range)	1100, 99 (819 to 1494)^g^	1118, 103 (819 to 1326)	1098, 97 (860 to 1494)
APOE genotype: %	^h^		
ε4‐carrier	29.7	60.8	22.9
Non‐carrier	70.3	39.2	77.1
Choice reaction time outcomes (unadjusted):			
RT (ms): mean, SD, (range)	781, 80, (615 to 1234)	787, 76, (615 to 1012)	778, 78, (623 to 1234)
Error rate (%): median, IQR, (range)	0, 0 to 4.5, (0 to 22.7)	0, 0 to 4.5, (0 to 18.2)	0, 0 to 4.5, (0 to 18.2)
Intra‐individual variability in RT: mean, SD, (range)	0.125, 0.034, (0.043 to 0.231)	0.133, 0.034, (0.043 to 0.201)	0.123, 0.033, (0.047 to 0.231)

Abbreviation: Aβ, amyloid beta; APOE, apolipoprotein E; IQR, interquartile range; MMSE, Mini‐Mental State Examination; RT, reaction time; SD, standard deviation.

a See section 2.2 and Lu et al.^31^ for definition.

b χ^2^, t tests, and rank‐sum tests were used to test for differences between the Aβ‐positive and ‐negative groups for the demographic, biomarker, and clinical variables; the only variable with a statistically significant difference was APOE (*P* < 0.0001).

c Age at assessment was calculated based on the date that the cognitive assessment was carried out (while assessments were typically completed on one day, 62 participants had to have their scans rescheduled for a later date, with a median interval of 49 days [range 1 to 216 days]).

d Z‐scores for childhood cognitive ability were based on the full National Survey of Health and Development cohort of N = 5362, so the mean for Insight 46 participants indicates that they had higher childhood cognitive ability on average than their peers not recruited to this substudy.

e n = 461 due to missing data.

f n = 454 due to missing data.

g n = 467 due to missing data.

h n = 500 due to missing data.

### 2.2 Life‐course and clinical variables

Childhood cognitive ability was measured at age 8 (or ages 11 or 15 if earlier data were missing) as a standardized z‐score based on tests of verbal and non‐verbal ability, as previously described.^31^

Educational attainment was represented as the highest qualification achieved by age 26, grouped into five categories: no qualification, below O‐levels (vocational), O‐levels and equivalents, A‐levels and equivalents, higher education (degree and equivalents).

Socioeconomic position was derived from participants’ own occupation at age 53, or earlier if this was missing, coded in six categories according to the UK Registrar General's Standard's Occupational Classification: unskilled, partially skilled, skilled manual, skilled non‐manual, intermediate, professional.

Participants were classified as having a neurological or psychiatric condition (including dementia and MCI) as previously described.^31^ Participants not meeting these criteria are hereafter referred to as cognitively normal and represent a sample free from possible confounding comorbidities.

### 2.3 Biomarker measures

As previously described,^32^, ^34^, ^38^ Aβ‐PET and multi‐modal MRI data were collected simultaneously during a 60‐minute scanning session on a single Biograph mMR 3T PET/MRI scanner (Siemens Healthcare, Erlangen), with intravenous injection of 370 MBq of 18F‐Florbetapir (Amyvid). Aβ deposition was quantified using the standard uptake volume ratio (SUVR) calculated from cortical regions of interest. A cut‐point for Aβ‐positivity was determined at SUVR >0.6104.^31^, ^34^, ^38^

Whole brain volume was generated from high resolution 3D T1‐weighted MRI using automated segmentation with manual editing.^32^ Total intracranial volume (TIV) was generated using statistical parametric mapping software (SPM12; http://www.fil.ion.ucl.ac.uk/spm).^32^ Global white matter hyperintensity volume (WMHV) was generated using an automated segmentation algorithm followed by visual quality control.^31^, ^34^

Apolipoprotein E (*APOE*) genotype was classified as ε4‐carrier or non‐carrier.^32^

### 2.4 Participants

Out of the 502 participants, 501 completed the CRT task. Of these, there were 406 cognitively normal participants with complete biomarker data, of whom 18% were Aβ‐positive^31^, ^34^ Of note, three participants had dementia and eleven met criteria for MCI. Participant characteristics are reported in Table 1.

### 2.5 Data analyses

The main analyses were performed using summary scores, as described below. See supporting information for analyses performed using trial‐by‐trial data, including comparison of arrow versus word stimuli, consideration of practice effects, and investigation of within‐subject speed‐accuracy trade‐offs. We combined outcomes across the two blocks as the magnitude of differences in RT and error rate between the two stimulus types was small (supporting information). Three outcomes were generated:

1. Mean RT for correct responses
2. Error rate (% incorrect responses)
3. Intra‐individual variability in RT (IIV) for correct responses, defined as SD/mean. This “coefficient of variation" is a commonly used measure that accounts for an individual's average RT (eg, Der and Deary^10^ and Hultsch et al.^17^), as variability is a function of overall response speed. The supporting information includes an illustration of IIV.

Two sets of analyses were conducted. To investigate demographic and life‐course predictors of performance on each outcome, all 501 participants were included to have as representative a sample as possible. Regression models were fitted (details below), with predictors of sex, age at assessment, childhood cognitive ability, education, socioeconomic position, and presence of a neurological or psychiatric condition (yes or no).

To investigate associations between task performance and biomarkers of brain pathologies, analyses only included participants classified as cognitively normal, and for whom complete biomarker data were available (n = 406). Regression models were fitted (details below), with predictors of amyloid status (positive or negative), whole brain volume, WMHV, and *APOE* genotype (ε4‐carrier or non‐carrier), in addition to the demographic and life‐course predictors included previously (sex, age at assessment, childhood cognitive ability, education, socioeconomic position). To adjust for the correlation between brain volumes and head size, TIV was also included as a covariate. Supplementary analyses were conducted to test whether SUVR was associated with differences in performance (see supporting information).

Mean RT was analyzed using a linear regression model. As a log‐transformation did not remove the skew, untransformed data were used for ease of interpretation, but bootstrapping was used to produce bias‐corrected and accelerated 99%, 95%, and 90% confidence intervals (CIs)from 2000 replications. Error rate was analyzed using a generalized estimating equations logistic regression model with an independent working correlation structure and robust standard errors to allow for correlation between repeated responses by the same participant. IIV was approximately normally distributed and was analyzed using a linear regression model. Speed‐accuracy trade‐offs were investigated by testing for Spearman's rank correlation between mean RT and error rate. All analyses were conducted using Stata‐15 (StataCorp). Statistical significance was set at *P* < 0.05.

## 3 RESULTS

The median duration of the task was 2.2 minutes (inter‐quartile range = 2.0 to 2.4). Descriptive statistics for CRT outcomes are reported in Table 1.

### 3.1 Speed‐accuracy trade‐offs

**FIGURE 2:**
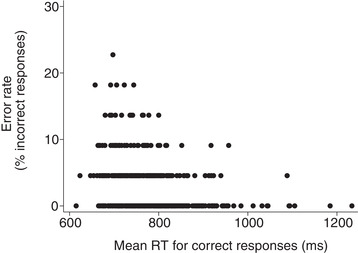
Error rate against mean reaction time (RT), showing speed‐accuracy trade‐off (n = 501)

Slower mean RT was associated with lower error rate, indicating a speed‐accuracy trade‐off (Spearman's ρ = −0.28, *P* < 0.0001; Figure 2). The correlation was weak due to a ceiling effect on accuracy. However, all of the participants with the highest error rates had relatively fast mean RTs, and all of the slowest‐responding participants made few errors.

### 3.2 Demographic and life‐course predictors

Results of the multivariable regression models are reported in Table 2.

**TABLE 2:** Associations between demographic and life‐course predictors and choice reaction time outcomes (n = 501)

Predictor	RT (ms): coefficient and 95% CIs	Odds ratio for making an error and 95% CIs	Intra‐individual variability in RT: coefficient and 95% CIs
Sex (female as reference)	−8.5 (−22.9, 7.8)	1.11 (0.85, 1.45)	−0.0050 (−0.0109, 0.0010)
Age at assessment (per year)	24.0^*^, ^†^ (14.7, 35.4)	0.81^*^ (0.68, 0.98)	0.0085^*^, ^†^ (0.0043, 0.0128)
Childhood cognitive ability (per z‐score)	−11.6^*^ (−23.2, −0.08)	0.73^*^, ^†^ (0.60, 0.89)	−0.0036 (−0.0082, 0.0009)
Education (per category)^‡^	−4.6 (−11.6, 1.6)	1.06 (0.92, 1.21)	−0.0030^*^ (−0.0058, −0.0002)
Socioeconomic position (per category)^‡^	−2.2 (−13.2, 6.3)	1.04 (0.91, 1.19)	0.0027 (−0.0005, 0.0059)
Neurological or psychiatric condition (cognitively normal as reference)^‡^	9.5 (−13.2, 40.3)	1.58^*^, ^†^ (1.14, 2.19)	0.0149^*^, ^†^ (0.0050, 0.0247)

Abbreviations: CI, confidence interval; IIV, intra‐individual variability; RT, reaction time.

* Significant at *P* < 0.05.

† Significant at *P* < 0.01.

‡ See section 2.2 for definitions.

Positive coefficients on RT reflect slower responses; odds ratios >1 for errors reflect higher error rates; positive coefficients on IIV reflect more variable responses. Multivariable regression models were used so each association is independent of all others.

Older age at assessment was associated with slower RT, lower error rate, and greater IIV.

Higher childhood cognitive ability was associated with faster RT and lower error rate, but did not show evidence of a statistically significant association with IIV.

Education was not associated with RT or error rate, but higher educational attainment was associated with slightly reduced IIV (equivalent to a 9.0% reduction between the lowest and highest categories of educational qualification [see section 2.2]).

Participants with neurological and psychiatric conditions did not differ from cognitively normal participants in terms of mean RT, but made more errors (adjusted mean error rates: 3.8% vs 2.5%) and had 12.0% greater IIV (adjusted means: 0.139 vs 0.124).

There was no evidence of sex differences on any outcome, nor any associations with socioeconomic position.

### 3.3 Associations with biomarkers

Results of the multivariable regression models are reported in Table 3.

**TABLE 3:** Associations between biomarkers and choice reaction time outcomes in cognitively normal participants (n = 406)

Predictor	Mean RT (ms): coefficient (95% CIs)	Odds ratio for making an error (95% CIs)	Intra‐individual variability in RT: coefficient (95% CIs)
Aβ status (negative as reference)	11.7 (−7.8, 30.8)	1.37 (0.93, 2.01)	0.013^*^, ^†^ (0.004, 0.021)
WMHV (per 10 mL)	−3.9 (−15.1, 7.6)	0.90 (0.69, 1.17)	−0.003 (−0.008, 0.003)
Whole brain volume (per 10 mL)	−1.7 (−3.6, 0.1)	1.01 (0.97, 1.04)	−0.000 (−0.001, 0.000)
*APOE*‐ε4 (non‐carriers as reference)	−2.2 (−18.0, 14.8)	0.94 (0.68, 1.30)	−0.004 (−0.011, 0.003)

Abbreviations: Aβ, amyloid beta; CI, confidence interval; IIV, intra‐individual variability; RT, reaction time; WMHV, white matter hyperintensity volume.

* Significant at *P* < 0.05.

† Significant at *P* < 0.01.

Positive coefficients on RT reflect slower responses; odds ratios >1 for errors reflect higher error rates; positive coefficients on IIV reflect more variable responses. Multivariable regression models were used so each association is independent of all others. In addition to the predictors listed, models also included sex, age at assessment, childhood cognitive ability, socioeconomic position, and total intracranial volume, but these associations are not reported as they are essentially unchanged from the first analysis (see Table 2).

**FIGURE 3:**
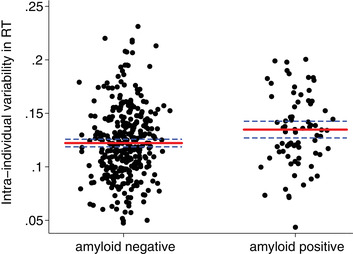
Intra‐individual variability in reaction time for cognitively normal amyloid‐beta negative (n = 332) and amyloid‐beta positive (n = 74) participants. Points show the raw intra‐individual variability (IIV) values. Solid red lines represent the adjusted means from the multivariate regression model (adjusted for whole brain volume, white matter hyperintensity volume, total intracranial volume, apolipoprotein E genotype, sex, age at assessment, childhood cognitive ability, education, and socioeconomic position), and the dashed blue lines represent their 95% confidence intervals. IIV is defined as the coefficient of variation (standard deviation/mean) for correct responses.

Aβ‐positive participants had 10.3% greater IIV than Aβ‐negative participants (adjusted means: 0.135 vs 0.122; Figure 3)—despite having similar mean RTs (see also Table 1). Aβ‐positive participants also had slightly higher error rates, but this difference was not statistically significant. Given the evidence that performance was influenced by speed‐accuracy trade‐offs, we conducted a post‐hoc analysis to investigate whether Aβ‐positive and ‐negative participants differed in their error rates after accounting for their overall response speed. The regression model for error rate was rerun with mean RT as an additional covariate, and results indicated that Aβ‐positive participants had higher odds of making an error after adjustment for their mean RTs (odds ratio [OR] = 1.48, 95% CI: 1.03 to 2.14, *P* = 0.03; adjusted mean error rates: Aβ‐positive = 3.3%; Aβ‐negative = 2.3% ms).

When dichotomous amyloid status was replaced with continuous SUVR, the same pattern emerged: SUVR was not associated with mean RT or error rate, but higher SUVR predicted greater IIV, with a steeper slope among Aβ‐positive participants (supporting information).

Whole brain volume, WMHV, and *APOE* genotype showed no evidence of associations with any outcome, although there was a trend toward an association between larger whole brain volume and faster RT (*P* < 0.1).

## 4 DISCUSSION

In this large population‐based sample of adults aged ≈70 years, we found that cognitively normal Aβ‐positive participants showed greater IIV in RT on a CRT task. To our knowledge, this has not been investigated before, but our results are consistent with a previous report of an association between CSF biomarkers of AD pathology and greater IIV on a test of task‐switching.^39^ We found no evidence that Aβ‐positivity was associated with slower RT. As expected, childhood cognitive ability and education showed some associations with performance, but there was no evidence of the sex differences reported by others.^10^, ^16^, ^19^, ^23^ Speed‐accuracy trade‐offs were identified as an important factor in interpreting response patterns.

### 4.1 Speed‐accuracy trade‐offs

The most error‐prone participants tended to have fast RTs, and the slowest‐responding participants tended to be highly accurate. Speed‐accuracy trade‐offs in CRT tasks have been observed in some^9^ but not all previous studies.^40^ We hypothesize that this difference may be explained by the precise experimental design, with easier tasks (such as ours and Nissan et al.^9^) being more susceptible to trade‐offs, and more complex tasks (eg, Vaportzis et al.^40^) tending to show positive correlations between speed and accuracy.

Within‐subject speed‐accuracy trade‐offs were also observed (supporting information), suggesting that errors tended to arise from hasty or anticipatory responses. There was evidence that individuals altered their speed‐accuracy strategy during the task, slowing down after making an error.

### 4.2 Demographic and life‐course predictors

#### 4.2.1 Age at assessment

The finding that older age was associated with slower and more variable RT is consistent with the literature.^9^, ^10^, ^11^, ^12^, ^13^, ^14^ However, it is perhaps surprising that this was seen in the Insight 46 cohort, because the age range was so narrow (69.2 to 71.8 years—reflecting the length of the data collection period, as participants were all born in the same week). Estimates from previous studies suggest a slowing of 2.0 to 3.4 ms per year across adulthood,^11^ or a decline of −0.096 SD per year.^14^ Our effect size of 24 ms per year, or 0.30 SD, is large in comparison. This result may in part reflect speed‐accuracy trade‐offs, as older age also predicted lower error rate; this is consistent with evidence that older people tend to respond more cautiously, prioritizing accuracy at the expense of speed.^41^, ^42^

We previously reported associations in Insight 46 between older age and poorer non‐verbal reasoning ability^31^ and smaller hippocampal volumes.^38^ As discussed previously, we considered the possibility that such effects may be partially explained by a recruitment bias whereby participants seen toward the beginning of the data collection period may have differed in some ways to those seen toward to the end. However, we found no objective evidence of differences in health or sociodemographic factors.

#### 4.2.2 Childhood cognitive ability, education, and socioeconomic position

Our results conform to previous analyses from the NSHD and Insight 46, which have identified childhood cognitive ability and education as key independent predictors of cognition across adulthood, whereas the effect of socioeconomic position is generally less important.^31^, ^43^

Consistent with extensive investigations into the relationship between RT and general cognitive ability^9^, ^24^, ^25^, ^26^, ^27^ higher childhood cognitive ability predicted faster RT more than 60 years later. This came at no cost to accuracy, suggesting that participants with higher cognitive ability may be better able to inhibit anticipatory responses. Conversely, educational attainment had no independent effect on RT and error rate, but was independently associated with smaller IIV.

#### 4.2.3 Sex differences

Contrary to some previous studies,^10^, ^16^, ^23^ in this large sample of narrow age range, and accounting for prior cognitive ability, there was no evidence of sex differences on any CRT outcome, although subtle effects may not be detectable over this small number of trials.

### 4.3 Associations with biomarkers

#### 4.3.1 Aβ pathology

The mean difference in IIV between Aβ‐positive and ‐negative participants (10.3%; equivalent to 0.37 SD) was of a similar magnitude to their difference on a non‐verbal reasoning test (0.39 SD), and greater than on the Preclinical Alzheimer Cognitive Composite (0.17 SD).^31^

As Aβ‐positive participants were all cognitively normal and more than a decade away from the estimated median age of onset of dementia in the UK,^44^ this raises the possibility that IIV may be a particularly sensitive measure of the cognitive consequences of accumulating AD pathology, whereas the general slowing of RT may perhaps occur at a later stage (as observed in individuals with MCI^16^, ^19^, ^20^, ^21^). The association between higher SUVR and greater IIV among Aβ‐positive participants further supports the potential utility of this measure. Follow‐up assessments of Insight 46 participants (currently underway) will provide an opportunity to test this.

There was a suggestion that Aβ pathology was associated with higher error rates, as Aβ‐positive participants made more errors than Aβ‐negative participants and this difference was statistically significant after adjustment for the trade‐off between RT and error rate. However, this finding needs to be interpreted with caution as this task was limited in its ability to measure subtle differences in accuracy due to the small number of trials (see section 4.4).

It is important to note that response speed and variability appear to be general markers of the integrity of the central nervous system, vulnerable to disruption across a range of neurodegenerative diseases and brain injuries, correlating with diverse physical health measures such as grip strength, forced expiratory volume, and waist circumference,^12^, ^45^, ^46^ and potentially affected by many common medications.^47^ Performance of CRT tasks is also subserved by multiple component executive processes including energizing, monitoring, and inhibiting.^36^ Further work is needed to investigate how changes in RT associated with AD pathology may relate to physical health measures and to other brain pathologies that accumulate with age.

#### 4.3.2 Brain volume and white matter disease

We previously reported that Insight 46 participants with smaller brain volumes and greater burden of white matter disease had slightly slower processing speeds on the Digit‐Symbol Substitution Test, a test which is particularly sensitive to overall brain health.^31^ We did not find evidence of similar effects on CRT, although associations were in the expected direction. As the detrimental effects of white matter disease on processing speed and RT are well established,^37^, ^48^, ^49^ the lack of an association between WMHV and CRT may be due to the fact that participants on the whole had very low levels of white matter disease.

### 4.4 Strengths and limitations

The CRT task typically took <3 minutes to complete, making it feasible for inclusion in a busy assessment schedule. However, the small number of trials limited the signal‐noise ratio and had a particular impact on the error rate measure, as a single incorrect response corresponded to a large proportionate increase in error rate. It is worth noting that many CRT studies place little focus on error rates, because RT is naturally the primary interest. Our findings suggest that errors on this task capture meaningful differences between individuals and are also inherently linked to differences in RT and IIV.

Strengths and limitations relating to the representativeness of Insight 46 participants have been previously discussed,^31^ the main limitations being that all participants are white and the sample is inevitably biased toward those in better health who are willing and able to travel to London and to have a brain scan. The absence of PET or cerebrospinal fluid measures of tau—limiting our ability to draw conclusions about the pathological basis for alterations in RT performance—will be addressed in future data collections.

### 4.5 Conclusions

In summary, this short CRT experiment provides novel evidence that cognitively normal older adults with Aβ pathology are, on average, less consistent in their response times. This builds on previous evidence that Aβ pathology is associated with subtle changes in multiple cognitive domains, even by age 70.

## FUNDING INFORMATION

This study is principally funded by grants from Alzheimer's Research UK (ARUK‐PG2014‐1946, ARUK‐PG2017‐1946), the Medical Research Council Dementias Platform UK (CSUB19166), and the Wolfson Foundation (PR/ylr/18575). The genetic analyses are funded by the Brain Research Trust (UCC14191). Florbetapir amyloid tracer is kindly provided by AVID Radiopharmaceuticals (a wholly owned subsidiary of Eli Lilly) who had no part in the design of the study. The National Survey of Health and Development is funded by the Medical Research Council (MC_UU_12019/06, MC_UU_12019/08). The funders of the study had no role in study design, data collection, analysis, interpretation, report writing, or in the decision to submit the article for publication.

## CONFLICTS OF INTEREST

Kirsty Lu reports no disclosures. Jennifer M. Nicholas reports no disclosures. Sarah‐Naomi James reports no disclosures. Thomas D. Parker is supported by a Wellcome Trust Clinical Research Fellowship (200109/Z/15/Z). Christopher A. Lane reports no disclosures. Ashvini Keshavan is supported by a Clinical Research Fellowship funded by the Wolfson Foundation. Sarah E. Keuss reports no disclosures. Sarah M. Buchanan reports no disclosures. Heidi Murray‐Smith reports no disclosures. David M. Cash is supported by an Alzheimer's Society Project Grant (AS‐PG‐205). Carole H. Sudre is supported by an Alzheimer's Society Junior Fellowship (AS‐JF‐17‐011). Ian B. Malone reports no disclosures. William Coath reports no disclosures. Andrew Wong reports no disclosures. Susie M.D. Henley reports no disclosures. Nick C. Fox is supported by the UCL/UCLH NIHR Biomedical Research Centre and the UK Dementia Research Institute at UCL. Marcus Richards reports no disclosures. Jonathan M. Schott is supported by the UCL/UCLH NIHR Biomedical Research Centre, UCL Hospitals Biomedical Research Centre, and Leonard Wolfson Experimental Neurology Centre and acknowledges the EPSRC (EP/J020990/1) and European Union's Horizon 2020 research and innovation programme (Grant 666992). Sebastian J. Crutch is supported by an Alzheimer's Research UK Senior Research Fellowship (ARUK‐SRF2013‐8).

## Supporting information

Figure S1. Distribution of reaction times for three participants with the maximum, median and minimum intra‐individual variability scores. *Each participant's reaction times to the individual stimuli are plotted (correct responses only). Intra‐individual variability (IIV) in RT is defined as the coefficient of variation (SD/mean). These three participants have been selected to illustrate the meaning of the IIV score and to put its magnitude in context: participant 1 has the maximum IIV, participant 2 has the median IIV and participant 3 has the minimum IIV. It can be seen that these three participants have similar mean RTs (774ms, 755ms and 763ms respectively) but the variability of their responses is very different*.

Supporting information.
