# Supplementary material for: Increased variability in reaction time is associated with amyloid beta pathology at age 70
Source: Alzheimers Dement (Amst). 2020 Aug 10;12(1):e12076. doi: 10.1002/dad2.12076 (PMC7416668; doi:10.1002/dad2.12076)
Supplement: Supplementary file 2 — Supporting information. [file DAD2-12-e12076-s002.docx]

# Supplementary Material

1. *Associations between SUVR and Choice Reaction Time (CRT) performance*

As described in section 2.5, analyses were rerun replacing dichotomised amyloid status with continuous SUVR to test whether increasing β-amyloid (Aβ) deposition was associated with differences in performance. For each outcome (mean RT, error rate, IIV) the models were first fitted across the full range of SUVR, and secondly using a linear spline with a knot at the cut-point for Aβ-positivity (SUVR > 0.6104), to explore whether the association differed for Aβ+ and Aβ- groups.

Results across the full range of SUVR were consistent with the analyses using dichotomised amyloid status: SUVR was not associated with mean RT (regression coefficient = 5.9 ms per 0.1 SUVR increment, *95% CIs* -3.4 to 15.9, *p* > 0.1) or error rate (*OR* = 1.09, *95% CIs* 0.87 to 1.36, *p* = 0.46), but higher SUVR predicted greater IIV (regression coefficient = 0.0062, *95% CIs* 0.0014 to 0.0110, *p* = 0.012). The spline analysis revealed that within Aβ+ and Aβ- groups separately, SUVR was not associated with performance on mean RT (Aβ+: regression coefficient = 4.7 ms per 0.1 SUVR increment, 95% CIs -13.9 to 23.4, *p* > 0.1; Aβ-: regression coefficient = 7.0, 95% CIs -15.3 to 29.4, *p* > 0.1) nor on error rate (Aβ+: *OR* = 1.16, 95% CIs 0.83 to 1.63, *p* = 0.38; Aβ-: *OR* = 1.01, 95% CIs 0.65 to 1.59, *p* = 0.95). Higher SUVR was associated with greater IIV among Aβ+ participants, but not among Aβ- participants (Aβ+: regression coefficient = 0.0112, 95% CIs 0.0016 to 0.0209, *p* = 0.023; Aβ-: regression coefficient = 0.0012, 95% CIs -0.0084 to 0.0108, *p* = 0.81).

1. *Trial-by-trial analyses*
   1. *Statistical models*

While the main analyses were conducted using summary outcome scores (see 2.5), trial-by-trial responses to each individual stimulus were analysed to investigate differences in reaction time (RT) and accuracy between Block 1 (arrow stimuli) and Block 2 (word stimuli), potential practice effects, and within-subject speed-accuracy trade-offs.

RTs were first log-transformed so that the distribution more closely approximated the normal distribution. RTs (correct responses only) were analysed using a generalized estimating equations (GEE) model, assuming a normal distribution for the dependent variable and an identity link (as with standard linear regression), but including an exchangeable correlation structure and robust standard errors to allow for the correlation between repeated measures of the same participant. The regression coefficients are quoted in exponentiated form as ratios for ease of interpretation; for example, a coefficient of 1.05 would mean that the factor was associated with 5% longer response time.

Response accuracy (correct *vs.* incorrect) was analysed using a GEE logistic regression model with an independent correlation structure and robust standard errors. Results are expressed as odds ratios for ease of interpretation.

All 501 participants were included in these supplementary analyses.

- 1. *Arrows vs. words*

The models for RT and accuracy described above were run with predictors of stimulus type (arrow *vs.* word), sex, age at assessment, childhood cognitive ability, education, socioeconomic position and presence of a neurological or psychiatric condition (yes *vs.* no). Results for the demographic and life-course predictors are not reported here as they were essentially unchanged from the main analyses (see 3.2)

On average, responses were 7% slower to words than arrows (95% CIs 1.06 to 1.07, *p* < 0.0001; adjusted means: words = 797 ms; arrows = 748 ms). The odds of making an error were 27% lower for words than arrows (95% CIs 0.59 to 0.90, *p* < 0.0001; adjusted means: words = 2.2% ms; arrows = 3.0%).

A possible explanation for the slower responses to word stimuli is that they place greater cognitive demands on the participant because they require higher-order processing of the concepts of left and right, whereas arrow stimuli simply require the participant to press the button on the same side as the arrow. A similar effect was found in a previous study which compared Choice RT to numbers and lights, and concluded that the slower responses to numbers could be attributed to the higher-order cognitive processing required (Nissan et al., 2013). One might expect that stimuli requiring greater cognitive processing (and consequently slower RT) would also elicit a higher error rate, as was the case in the study of numbers and lights*.* Our finding of the opposite pattern (a higher error rate for arrows) implies a within-subject speed-accuracy trade-off (see d)).

- 1. *Practice effects*

As the arrow stimuli (Block 1) always preceded the word stimuli (Block 2), comparison between them could be confounded by practice effects. While this cannot be tested explicitly, exploring practice effects within the two blocks separately could give an indication of whether practice effects are generally observed on this test. Practice effects on RT and error rate were investigated by rerunning the models for RT and accuracy (see a)) in each block separately, with an additional factor of trial number (1 to 11).

On average, RT slightly decreased during the arrow block (regression coefficient = 0.996 per successive trial, *95% CIs* 0.995 to 0.997, *p* < 0.0001) suggesting a practice effect, but slightly increased during the word block (regression coefficient = 1.002, *95% CIs* 1.001 to 1.003, *p* < 0.0001). This difference could be due to the fact that the arrow block came first so perhaps participants were still getting used to the task. There was no evidence of statistically significant practice effects in error rate (Arrow block: *OR* = 1.004, *95% CIs* 0.998 to 1.094, *p* = 0.063; Word block: *OR* = 0.987, *95% CIs* 0.932 to 1.045, *p* = 0.656). Overall it does not appear that performance was strongly influenced by practice effects.

- 1. *Within-subject speed-accuracy trade-offs and post-error slowing*

Incorrect responses were faster than correct responses on average (621ms *vs.* 783 ms), implying a within-subject speed-accuracy trade-off (i.e. participants were more likely to make an error when they responded hastily.) To investigate this in more detail, the regression model for the odds of making errors (see a)) was rerun with RT included as an additional factor, to investigate whether the speed of a response predicted whether that response would be correct or incorrect. RT was not log transformed for this analysis because it was included as a predictor rather than as the outcome and retaining the original scale aided interpretability.

Results showed that errors were less likely to occur with increasing RT, with a 2% reduction in the odds of making an error per additional millisecond (*OR* = 0.98, *95% CIs* 0.98 to 0.98, *p* < 0.0001). With RT included in the model, the difference in error rate between word and arrow stimuli was reversed such that word stimuli were associated with greater odds of an error (adjusted error rates: words 3.9% *vs* arrows 2.0%, *OR* = 2.16, *95% CIs* 1.68 to 2.78, *p* < 0.0001). This suggests that the earlier result of a higher error rate for arrows can be fully accounted for by speed-accuracy trade-offs, since responses to arrows were faster (see b)).

Individuals may alter their speed-accuracy strategy during a task: after making an error they may shift their strategy to place an increased priority on accuracy (“post-error slowing”), whereas after a run of correct responses they may shift their strategy to place an increased priority on speed. Toggling between the two competing priorities is a legitimate strategy for maximising both over the course of a task (Dang et al., 2018). Such alterations in strategy would clearly affect intra-individual variability (IIV) in RT, and may explain why some studies have reported that IIV (for correct responses) is a function of error rate (Der & Deary, 2006; Hultsch et al., 2000). To investigate whether Insight 46 participants showed evidence of post-error slowing, all correct responses were classified as either “post-correct” or “post-error”, according to whether the response immediately preceding them was correct or incorrect. As expected, “post-error” responses were slower than “post-correct” responses (mean RTs of 846 and 779 ms, respectively). The regression model for RT (see a)) was rerun including this binary factor as an additional predictor, and the results confirmed a statistically significant effect of post-error slowing: post-error responses were 14% slower on average (95% CIs 1.12 to 1.16, *p* < 0.0001) after adjustment for stimulus type (arrow *vs.* word) and the demographic and life-course predictors listed earlier. The impact of this post-error slowing on IIV is explored further below.

1. *Intra-individual variability (IIV)*

**Figure S1** illustrates how the IIV score is derived from the underlying response times.

The phenomenon of post-error slowing means that IIV is likely to be higher for those who make more errors (Der & Deary, 2006; Hultsch et al., 2000). To investigate the extent to which intra-individual variability in RT could be predicted from error rate on this task, the regression model for IIV (see 2.5) was rerun with error rate included as an additional factor. As expected, higher error rate was associated with greater IIV (regression coefficient = 0.0020 per percentage point increase in error rate, *95% CIs* 0.0013 to 0.0028, *p* < 0.0001). However, error rate did not explain the associations between IIV and the predictors reported in the main analyses (educational attainment, presence of a neurological or psychiatric condition, and amyloid status – see 3.2 and 3.3) as these associations were essentially unchanged when adjusting for error rate (regression coefficient for education = -0.0030, 95% CIs -0.0060 to -0.0005, *p* = 0.019; regression coefficient for neurological or psychiatric condition = 0.0120, 95% CIs 0.0024 to 0.0217, *p* = 0.015; regression coefficient for amyloid status = 0.0112, 95% CIs 0.0026 to 0.0198, *p* = 0.011).

# References

Dang JS, Figueroa IJ, Helton WS. You are measuring the decision to be fast, not inattention: the Sustained Attention to Response Task does not measure sustained attention. Exp Brain Res 2018:1–8. https://doi.org/10.1007/s00221-018-5291-6.

Der G, Deary IJ. Age and sex differences in reaction time in adulthood: results from the United Kingdom Health and Lifestyle Survey. Psychol Aging 2006;21:62–73. https://doi.org/10.1037/0882-7974.21.1.62.

Hultsch DF, MacDonald SWS, Hunter MA, Levy-Bencheton J, Strauss E. Intraindividual variability in cognitive performance in older adults: comparison of adults with mild dementia, adults with arthritis, and healthy adults. Neuropsychology 2000;14:588–98. https://doi.org/http://dx.doi.org/10.1037/0894-4105.14.4.588.

Nissan J, Liewald D, Deary IJ. Reaction time and intelligence: Comparing associations based on two response modes. Intelligence 2013;41:622–30. https://doi.org/10.1016/J.INTELL.2013.08.002.
